# Mitochondrial genetics

**DOI:** 10.1093/bmb/ldt017

**Published:** 2013-06

**Authors:** Patrick Francis Chinnery, Gavin Hudson

**Affiliations:** Institute of Genetic Medicine, International Centre for Life, Newcastle University, Central Parkway, Newcastle upon Tyne NE1 3BZ, UK

**Keywords:** mitochondria, genetics, mitochondrial DNA, mitochondrial disease, mtDNA

## Abstract

**Introduction:**

In the last 10 years the field of mitochondrial genetics has widened, shifting the focus from rare sporadic, metabolic disease to the effects of mitochondrial DNA (mtDNA) variation in a growing spectrum of human disease. The aim of this review is to guide the reader through some key concepts regarding mitochondria before introducing both classic and emerging mitochondrial disorders.

**Sources of data:**

In this article, a review of the current mitochondrial genetics literature was conducted using PubMed (http://www.ncbi.nlm.nih.gov/pubmed/). In addition, this review makes use of a growing number of publically available databases including MITOMAP, a human mitochondrial genome database (www.mitomap.org), the Human DNA polymerase Gamma Mutation Database (http://tools.niehs.nih.gov/polg/) and PhyloTree.org (www.phylotree.org), a repository of global mtDNA variation.

**Areas of agreement:**

The disruption in cellular energy, resulting from defects in mtDNA or defects in the nuclear-encoded genes responsible for mitochondrial maintenance, manifests in a growing number of human diseases.

**Areas of controversy:**

The exact mechanisms which govern the inheritance of mtDNA are hotly debated.

**Growing points:**

Although still in the early stages, the development of *in vitro* genetic manipulation could see an end to the inheritance of the most severe mtDNA disease.

## Mitochondria

The mitochondrion is a highly specialized organelle, present in almost all eukaryotic cells and principally charged with the production of cellular energy through oxidative phosphorylation (OXPHOS). In addition to energy production, mitochondria are also key components in calcium signalling, regulation of cellular metabolism, haem synthesis, steroid synthesis and, perhaps most importantly, programmed cell death (apoptosis).^1^ However, the simplistic elegance of biochemical ATP production belies a, complex, synergistic relationship between two genomes: the mitochondrial genome (mtDNA) and the nuclear genome (nDNA). The aim of this review is to introduce these two genomes and shed light on the clinical problems arising when communication breaks down. The emphasis is on the basic science underpinning mitochondrial diseases. Clinical aspects are not considered in detail because they have recently been reviewed elsewhere in open-access publications.^2–4^

## mtDNA

**Fig. 1:**
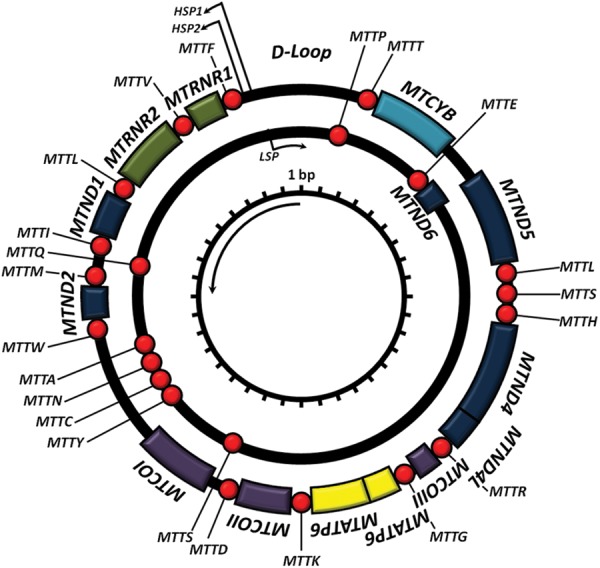
Mitochondrial DNA. Schematic diagram of the 16.6-kb, circular, double-stranded mtDNA molecule, where the outer circle represents the heavy strand and the inner circle the light strand. Shown are the genes encoding the mitochondrial RC: *MTND1–6*, *MTCOI–II*, *MTATP6 and 8* and *MTCYB*; the two ribosomal RNAs (green boxes) and each of the 22 tRNAs (red spheres).

MtDNA is the only source of critical cellular proteins outside of the eukaryotic nucleus. In the majority of eukaryotes, mtDNA is organizsed as a circular, double-stranded DNA molecule (Fig. 1).^5^ The strands are distinguished by their nucleotide composition: heavy (H-strand) is guanine rich, compared with the cytosine-rich light strand (L-strand). The length varies between species (15 000–17 000 bp), but is fairly consistent in humans (∼16 569 bp).^5^ MtDNA is a multi-copy DNA, with cells containing between 100 and 10 000 copies of mtDNA (dependent upon cellular energy demand).

### Structure

MtDNA contains 37 genes, 28 on the H-strand and 9 on the L-strand. Thirteen of the genes encode one polypeptide component of the mitochondrial respiratory chain (RC), the site of cellular energy production through OXPHOS. Twenty-four genes encode a mature RNA product: 22 mitochondrial tRNA molecules, a 16 s rRNA (large ribosomal subunit) and a 12 s rRNA (small ribosomal subunit).^5^ Unlike its nDNA counterpart, mtDNA is extremely efficient with ∼93% representing a coding region. Unlike nDNA, mtDNA genes lack intronic regions and some genes, notably *MTATP6* and *MTATP8*, have overlapping regions. Most genes are contiguous, separated by one or two non-coding base pairs. mtDNA contains only one significant non-coding region, the displacement loop (D-loop).^5^ The D-loop contains the site of mtDNA replication initiation (origin of heavy strand synthesis, OH) and is also the site of both H-strand transcription promoters (HSP1 and HSP2).

The mitochondrial genetic code differs slightly from nuclear DNA (nDNA). MtDNA uses only two stop codons: ‘AGA’ and ‘AGG’^6^ (compared with ‘UAA’, ‘UGA’ and ‘UAG’ in nDNA), conversely ‘UGA’ encodes tryptophan. To compensate UAA codons have to be introduced at the post-transcriptional level. In addition ‘AUA’, isoleucine in nDNA, encodes for methionine in mtDNA.

### Inheritance

Prevailing theory suggests that mtDNA is maternally inherited, with mtDNA nucleoids the unit of inheritance. During mammalian zygote formation, sperm mtDNA is removed by ubiquitination, likely occurring during transport through the male reproductive tract.^7^ Consequently, the mtDNA content of the zygote is determined exclusively by the previously unfertilized egg.

To date only a single case of paternal transmission in humans has been recorded.^8^ However, paternal transmission in other animals is both common and recurring. Theory suggests that the lack of paternal inheritance is due to either (i) a dilution effect; sperm contain only 100 copies of mtDNA, compared with 100 000 in the unfertilized egg, (ii) selective ubiquitination of paternal mtDNA or (iii) the ‘mtDNA bottleneck’ excludes the ‘minor’ paternal alleles.^7^ The advent of deep, next generation sequencing, allowing mtDNA can be sequenced at great depths (>20 000 fold) may enable researchers to revisit this phenomenon.

### Homoplasmy and heteroplasmy

Cells contain thousands of molecules of mtDNA;^9^ and in the majority of cases their sequence is identical, known as homoplasmy. However, an inefficient mtDNA repair, a localized oxidative environment and increased replication^10^ make mtDNA mutation frequent. The polyploid nature of mtDNA means that mutations often co-exist with their wild-type counterpart in various proportions (termed heteroplasmy). The proportion of mutant has important consequences in understanding mitochondrial disease (discussed later).^11^

## nDNA and mitochondrial function

**Fig. 2:**
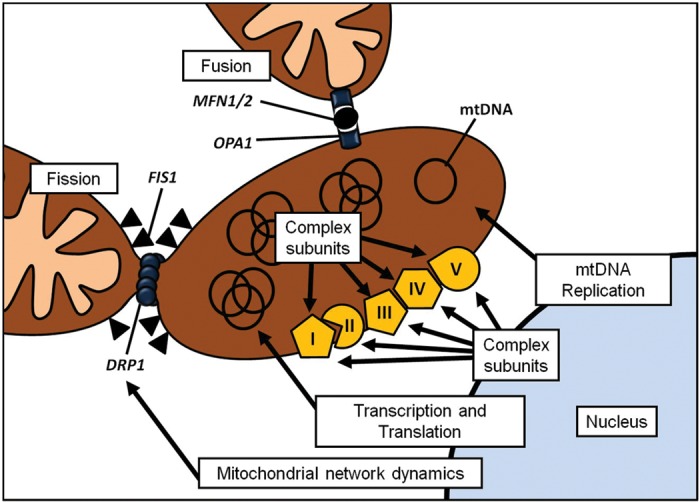
Interaction between nDNA and mtDNA. Cartoon demonstrating the complex interaction between genes encoded by nDNA and the processes they control in the mitochondrion.

According to recent data the mitochondrial proteome is estimated at ∼1500 proteins.^12^ Mitochondria are dependent upon the nuclear genome for the majority of the OXPHOS system and also for maintaining and replicating mtDNA as well as organelle network proliferation and destruction (Fig. 2).

### OXPHOS system

To date, 92 structural OXPHOS subunit genes have been identified: 13 encoded by mtDNA (Fig. 1) and 79 encoded by the nuclear genome. Briefly, complex I (NADH:ubiquinone oxidoreductase), the largest of the RC components, consists of 44 subunits: 14 enzymatic ‘core subunits’ (7 from mtDNA and 7 from nDNA)^13^ and a further 30 nDNA accessory subunits thought to maintain complex stability.^14^ Complex II (succinate:ubiquinone oxidoreductase) is encoded entirely by nDNA (four subunits). Complex III (ubiquinol:cytochrome *c* oxidoreductase) contains 11 subunits, 1 encoded by mtDNA (*MTCYB*) and 10 encoded by nDNA.^15^ Complex IV (cytochrome *c* oxidase) consists of three mtDNA-encoded subunits and a further 11 nDNA-encoded subunits. Finally, complex V (F_0_F_1_-ATP synthase) comprises 19 subunits, 2 encoded by mtDNA and the remaining 17 encoded by nDNA.

In addition, nDNA encodes over 35 proteins required for the RC assembly: complex I = 11 nDNA assembly factors,^16^ complex III = 2,^15^ complex IV = 18^17^ and complex V = 4.^18^

### mtDNA replication

Unlike nDNA, mtDNA replication is not governed by the cell cycle (eukaryotic cell division) and is continuously recycled. MtDNA replication and integrity maintenance is handled entirely by the nDNA. In eukaryotes, mtDNA is replicated in a ‘replisome’ (a DNA/protein complex making up the replication machinery) by a trimeric protein complex composed of a catalytic subunit: polymerase gamma, a 140 kDa DNA polymerase encoded by *POLG* and two 55 kDa accessory subunits, encoded by *POLG2*.^19^ This enzyme complex performs three activities, DNA polymerase activity, 3′-5′ exonuclease/proofreading activity and a 5′dRP lyase activity (required for enzymatic DNA repair).

In addition, the replisome also includes the mitochondrial single-stranded binding protein (encoded by *mtSSB*), which is involved in stabilizing single-stranded regions of mtDNA at replication forks, enhancing polymerase gamma activity. Twinkle is a 5′-3′ DNA helicase, which unwinds double-stranded mtDNA, facilitating mtDNA synthesis, as well as acting as a mtDNA primase (an enzyme required to prime nucleotide synthesis).^19^ Several topoisomerases have been indentified in humans, including the mitochondrial topoisomerases 1 (encoded by *TOP1mt*) and IIIα (encoded by *TOP3a*). Finally, the synergy between mitochondrial transcription factor A (encoded by *TFAM*) and mtDNA copy number suggests that TFAM may act as an mtDNA chaperone (a protein that assists the function of another protein) protecting it against oxidative damage.

### mtDNA arrangement

Like its nDNA counterpart, mtDNA is also packaged in protein–DNA complexes, known as nucleoids.^20^ MtDNA nucleoids are associated with the inner mitochondrial membrane, spaced evenly along the cristae. In addition to a single mtDNA molecule,^21^ mtDNA nucleoids contain a number of proteins.^20^ Principally the site of mtDNA replication, it is unsurprising that mtDNA nucleoids contain the protein machinery required for DNA replication, transcription, repair and packaging, including the mtDNA polymerase *POLG*, its accessory subunit *POLG2*, the activator of mtDNA transcription (encoded by *TFAM*) as well as mtDNA helicases and binding proteins (*twinkle* and *mtSSB*, respectively).^20^ In addition, mtDNA nucleoids contain chaperone proteins (HSP90-β and HSP70) required for mtDNA stability.

### Transcription and translation

Transcription of mtDNA is ‘prokaryotic like’ and was thought of a two-component system involving a protein complex containing the mitochondrial RNA polymerase (*POLRMT*) and two transcription factors (TFB1M and 2M).^22,23^ However, recent research indicates that TFB1M does not modulate mtDNA transcription in the presence of TFB2M, rather it acts as a dimethyltransferase which stabilizes the small subunit of the mitochondrial ribosome. RNA transcription is regulated by mitochondrial transcription factor A (*TFAM*).^24^

Briefly, each strand is transcribed as a polycistronic precursor mRNA molecule (i.e. the mRNA contains all of the genes in one molecule). Light-strand transcription is initiated from the light-strand promoter; however, heavy-strand transcription initiates from two heavy strand promoters: HSP1 and HSP2 (Fig. 1).^25^ Transcript elongation is performed by *POLRMT*, enhanced by both ‘transcription elongation factor mitochondrial’ (*TEFM*) and termination of mature transcripts is carried out by mitochondrial termination factor 1 (*MTERF1*).^25^

Translation of the 13 mtDNA protein coding genes occurs in the mitochondria. The mitoribosomes are partly coded by mtDNA (*MTRNR1* and *MTRNR2*, Fig. 1), but require a further 81 nDNA proteins. Translation is initiated by two mitochondrial initiation factors: mtIF1 and mtIF3.^26,27^ mtIF3 begins initiation by dissociating the ‘mitoribosome’ (the mitochondrial ribosomes) allowing assembly of the initiation complex.^28^ MRNA is then bound to the small subunit, aligning the start codon to the peptidyl site of the mitoribosome. Peptide elongation is controlled by a number of nuclear-encoded genes, including mitochondrial elongation factor Tu (mtEFTu),^29,30^ which binds the tRNA to the mitoribosome and mitochondrial elongation factor G1 (mtEFG1), required to move the newly added amino acid along one position and allowing amino acid inclusion.^31^ Translation termination is carried out solely by mitochondrial release factor 1a (mtRF1a),^32^ which recognizes the stop codons (UAA and UAG)^33^ and triggers hydrolysis of the bond between the terminal tRNA and the nascent peptide.

### Controlling mitochondrial network dynamics

Mitochondria are often depicted as distinct organelles; however, they actually form a complex reticulum that is undergoing continual fusion and fission (Fig. 2).^34^ It is likely that fusion has evolved as a mechanism to promote intermictochondrial cooperation—allowing the sharing and dissemination of mtDNA and mitochondrial proteins. Fission promotes mitochondrial compartmentalization,^34^ a mechanism that is needed to distribute mitochondria during cell division. Mitochondrial network dynamics, much like mtDNA replication, is controlled completely by nDNA, although likely involves mtDNA–nDNA communication.^34^

#### Mitochondrial fusion

The principle player in mitochondrial fusion is mitofusin (Mfn) and mammalian mitochondria contain two similar mitofusin proteins: Mfn1 and Mfn2 (Fig. 2),^34^ sharing 80% sequence homology. Studies indicate that both Mfn1 and Mfn2 uniformly localize to the mitochondrial outer membrane and overexpression leads to peri-nuclear clustering on mitochondria.^34^ Mitochondrial fusion is also dependent upon OPA1 expression (Fig. 2),^34^ where inhibition of gene expression causes an increase in mitochondrial fragmentation, conversely the overexpression of *OPA1* breaks the network into spheres.

#### Mitochondrial fission

*DNM1L*, dynamin 1 like, controls mitochondrial fission in mammalian cells (Fig. 2).^34^ *DNM1L* codes for a principally cytosolic protein; however, it also localizes to fission sites on the mitochondria. Similar to Mfn1, the overexpression of ‘mutant’ *DNM1L* results in a breakdown of mitochondrial networks. Due to its dynamin similarity, two different functions have been proposed for *DNM1L*. It has been hypothesized that *DNM1L* may mechanically mediate membrane fission through GTP hydrolysis; alternatively, it may act as a signalling molecule, conscripting and activating separate fission enzymes such as Dnm1: the yeast homologue of Drp1.

## Areas of agreement

### Mitochondrial disease

**Fig. 3:**
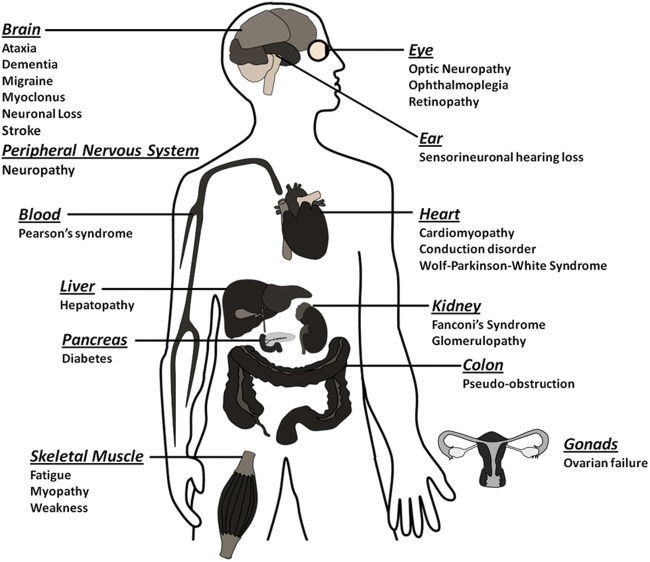
Clinical spectrum of mitochondrial disease. Schematic diagram showing the organ and corresponding disease affected by mitochondrial dysfunction.

An area where all mitochondrial researchers would agree is the capacity for mitochondrial dysfunction to manifest as disease. Mitochondrial disease is principally a chronic loss of cellular energy, where a failure to meet cellular energy demand results in a clinical phenotype. The clinical spectrum of mitochondrial disease is diverse (Fig. 3); however, tissues where there is a high metabolic demand, such as the central nervous system (CNS) or heart, are typically affected.

The broad clinical spectrum of mitochondrial dysfunction, coupled with the heterogeneity of mtDNA variation, makes the prevalence of mitochondrial DNA (mtDNA) difficult to calculate. Estimates, based on clinical observations, indicate that as many as 1 in 5000 people in the North East of England have manifested mitochondrial disease,^35^ with similar figures reported in other parts of the world.^36–38^

### Identifying and diagnosing mitochondrial genetic disease: general principles

Mitochondrial dysfunction should be considered in the differential diagnosis of any progressive, multisystem, disorder. However, clinical diagnosis can be difficult if patients do not present with ‘classical mitochondrial’ disease (see later).

A detailed family history is important; a clear maternal inheritance (without male transmission) indicates a primary mtDNA defect, whilst an autosomal inheritance pattern is indicative of nDNA interaction. In many cases blood and/or CSF lactate concentration,^39^ neuroimaging,^40,41^ cardiac evaluation and muscle biopsy for histological or histochemical evidence can indicate mitochondrial disease. However, establishing a molecular genetic diagnosis is preferred.

Molecular genetic testing can be carried out on DNA extracted from blood (useful for the identification of some mtDNA and nDNA mutations),^42,43^ but DNA extracted from the affected tissue is preferred (as pathogenic mtDNA mutations are often not detectable in blood).^44^ Southern blot analysis can be used to identify mtDNA rearrangements and ‘common’ mutations can be targeted by Sanger sequencing of either mtDNA or nDNA.

## The genetics of mitochondrial disease

The complex interaction between the two cellular genomes means mitochondrial disease can arise through either (i) a primary mtDNA defect or (ii) a defect in a nuclear-encoded mitochondrial protein.

### mtDNA and disease

#### Understanding mtDNA variation

mtDNA integrity is constantly attacked by mitochondrial reactive oxygen species (ROS) generated during cellular OXPHOS.^45^ ROS are potent genotoxic agents, which cause mutagenic and cytotoxic effects. The proximity of mtDNA to the site of mitochondrial ROS production (principally complexes I and III of the RC) is the major cause of oxidative lesions and mtDNA instability and is directly responsible for the higher nucleotide instability when compared with nDNA.

Despite being packaged in mitochondrial nucleoids and possessing DNA repair pathways evolved to cope with oxidative damage, including base excision repair mechanisms,^46^ mtDNA mutation rates are significantly higher than nDNA. Mutation creates two distinct classes of mtDNA variant: (i) single-base-pair variants and (ii) mtDNA rearrangements (deletions and insertions). Single-base-pair variants are typically inheritable and are either common in the populace (as proposed neutral variants) or enriched in individuals with disease (as mtDNA mutations). Understanding the complex nature of mtDNA variation is critical to understanding its affect on disease and there are a few key points that must be understood before assessing an mtDNA variant.

#### Consequences of mtDNA heteroplasmy

MtDNA heteroplasmy (described earlier) has a complex relationship with disease. The clinical expression of a heteroplasmic pathogenic mtDNA mutation is directly correlatable with the relative proportion of wild-type and mutant genomes.^47^ For common point mutations, a typical threshold of 80–90% mutant is required to manifest as disease at the cellular level,^48,49^ and tissue levels correlate loosely with the severity of the clinical phenotype. However, there is emerging evidence that mutation levels can change over time, increasing in post-mitotic tissues, such as brain and muscle and decreasing in mitotic tissues including blood. This can present a challenge when interpreting some clinical molecular genetic tests.^44,50,51^

#### Common mtDNA variation

Evolutionarily, common inherited mtDNA mutations have created stable population subgroups separated by common sequence variation known as haplogroups. Many of the major sub-divisions occurred over 10 000 years ago, developing as humans migrated into new geographic areas. Over 95% of Europeans belong to 1 of 10 major haplogroups, H, J, T, U, K (a subgroup of U), M, I, V, W and X, with each haplogroup defined by specific sequence variants within the population.^52^ These common, inherited, mtDNA variants are usually not heteroplasmic, and due to their selection neutrality have become fixed in the population. However, different haplogroups have been associated with a variety of human diseases, including primary mitochondrial disorders such as Leber's hereditary optic neuropathy (LHON, an age-related loss of vision), where background mitochondrial haplogroup has a direct, functional, effect on the RC protein complex assembly;^53^ but has expanded to include age-related neurodegenerative disorders such as Parkinson's disease (PD)^54^ Alzheimer's disease^55,56^ and age-related macular degeneration.^57^

#### Rare mtDNA variation

Rare, inherited, point mutations are a major cause of disease in humans, with an estimated incidence of 1 in 5000.^58^ They primarily occur in protein coding and tRNA genes and ultimately result in a reduction of cellular energy, through either a reduction in mitochondrial RC enzyme activity or an impairment of mitochondrial protein synthesis.^59^ Unlike common inherited variants, rare point mutations are often heteroplasmic.

In contrast to point mutations, primary mitochondrial rearrangements of mtDNA are not inheritable; they are primarily, sporadic, large-scale deletions, typically heteroplasmic and usually result in disease. To date around 120 different mtDNA deletions have been identified in patients with mitochondrial disease.^60^ Similarly to mtDNA point mutations, the ratio of deleted versus ‘wild-type’ molecules is critical to disease aetiology, with mtDNA deletions manifesting disease at a lower heteroplasmic threshold (∼50–60%).^61^ The exact mechanism of deletion formation is under debate and current research indicates two likely models of deletion formation: (i) replication error and (ii) mtDNA repair inefficiency.^62,63^

#### ‘Classical’ mtDNA diseases

LHON is a common cause of inherited blindness that typically presents with bilateral, painless, sub-acute visual failure in young adult males. LHON was the first maternally inherited disease to be associated with an mtDNA point mutation.^64^ Today, clinical diagnosis is usually confirmed by molecular genetic analysis for one of three ‘common’ mtDNA mutations, which all affect genes coding for complex I subunits of the RC: m.3460G>A, m.11778G>A and m14484T>C.^65^ Mitochondrial dysfunction causes a specific loss of retinal ganglion cells,^66^ whilst preserving the remaining retinal layers. The optic nerve also shows characteristic degeneration and an accumulation of mitochondria suggesting an impairment of axoplasmic transport. LHON mutations are typically homoplasmic; however, not all patients harbouring a pathogenic LHON mtDNA mutation develop visual failure. Studies of LHON have identified common mtDNA variants that may modulate LHON expression;^67,68^ additionally environmental factors, such as cigarette smoke^69^ and oestrogen levels may play a role.^70^ However, the majority of research has focused on the identification of a nuclear-encoded susceptibility allele.^67,71–74^

Non-syndromic and aminoglycoside-induced sensorineuronal hearing loss is associated with m.1555A>G, a homoplasmic point mutation in the 12sRNA gene.^75^ The variant alters a highly conserved region of 12sRNA, mutating the molecule to more closely resemble its bacterial homologue. *In vitro* experiments on m.1555A>G mutant cell lines demonstrated that exposure to aminoglycoside would impair growth; however, not all symptomatic individuals have been exposed to aminoglycoside.^75^

Surprisingly, given that they make up only 5% of mtDNA, the vast majority of pathogenic mtDNA point mutations occur in the tRNA genes (Fig. 1).^76,77^ In addition, pathogenic tRNA mutations are typically heteroplasmic.

Mitochondrial encephalomyopathy, lactic acidosis and stroke-like episodes (MELAS) is typically a childhood, multisystem disorder. Patients commonly manifest with generalized tonic-clonic seizures, recurrent headaches, anorexia with recurrent vomiting and postlingual hearing loss,^78–80^ but can manifest with impaired: motor ability, vision and mental acuity due to the cumulative effect of multiple stroke-like episodes. MELAS is commonly (80% of cases) caused by a A>G transition at m.3243 in *MTTL1*,^81^ but is also associated with variants in *MTND5*.^82^ Biochemically, MELAS manifests as defects of complex I and IV activity; however, care must be taken when interpreting the findings as biochemical results can often appear normal.

Myoclonus epilepsy with ragged red fibres (MERRF) is a neuromuscular disorder primarily caused by m.8344A>G in *MTTK*.^83^ Clinically, patients with m.8344A>G present with myoclonus, epilepsy, muscle weakness, cerebellar ataxia and dementia, although neurological symptoms can develop with age.^83^ Clinical severity is correlated with patient heteroplasmy with high levels of mutant mtDNA often causing, severe complex I or IV deficiency and occasionally a combined complex I and IV deficiency. Much like MELAS, the genotype–phenotype correlation of m.8344A>G can be extended beyond MERRF. M.8344A>G has been identified is diverse mitochondrial phenotypes such as Leigh's syndrome.

m.7472insC, affecting *MTTS* (Fig. 1), was first identified in a large Italian family presenting with hearing loss, ataxia and myoclonus. This mutation was later found in several unrelated families, all showing a wide clinical spectrum, including isolated hearing loss, ataxia and MERRF. This mutation has been found at increasing frequencies in families presenting with maternally inherited hearing loss.

Pathogenic rearrangements of mtDNA are typically large-scale deletions and to date over 120 different pathogenic mtDNA deletions have been identified.^60^ As described previously, mtDNA deletions are typically sporadic and not inheritable. Clinical severity is directly correlatable with the level and tissue distribution of the rearrangement and mitochondrial dysfunction is simply a result of the removal of key mitochondrial genes. Homoplasmic tRNA gene loss is particularly detrimental as mitochondria cannot synthesize a functional OXPHOS system. mtDNA deletions are associated with three main clinical phenotypes: Kearns–Sayre syndrome (KSS),^84^ sporadic progressive external ophthalmoplegia (PEO)^85^ and Pearson's syndrome.^86^

KSS is an early onset, sporadic, disorder characterized by PEO and pigmentary retinopathy; however, cases can also present with cerebellar syndrome, heart block, diabetes and shortness of stature. Mitochondrial dysfunction manifests as ragged red fibres (RRFs), an accumulation of dysfunctional mitochondria in the sub-sarcolemmal region of a muscle fibre (detectable when a muscle section is stained with Gomori trichrome stain).^85^

Large-scale deletions and duplications of mtDNA are a known cause of Pearson's bone-marrow–pancreas syndrome, a rare infant disorder characterized by infantile sideroblastic anaemia and occasionally including severe exocrine pancreatic insufficiency.^86^

### nDNA variation and mitochondrial disease

Nuclear–mitochondrial disease can be classified into four distinct groups: (i) disorders resulting from a reduction in mtDNA stability; (ii) disorders resulting from mutations in nuclear-encoded components or assembly factors of the OXPHOS system; (iii) disorders resulting from mutations affecting mitochondrial translation and (iv) disorders due to defects in genes controlling mitochondrial network dynamics.

#### Disorders resulting from a reduction in mtDNA stability

A growing number of disorders have become associated with mtDNA instability, primarily a result of impaired mtDNA replication. Mutations in *POLG*, the gene encoding the only mtDNA polymerase, are by far the commonest cause of mtDNA stability disorders. Mutations in the *POLG* gene can cause either point mutations (through impaired mtDNA proofreading) or deletions (through impaired polymerase activity) in mtDNA.^19^ The first pathogenic mutations in *POLG* were identified in families with autosomal dominant PEO (adPEO); however, the spectrum of disease associated with *POLG* mutations has been expanded to include autosomal recessive PEO, adult onset ataxia, Alpers' syndrome, parkinsonism and premature ovarian failure.^87^

adPEO, characterized by multiple mtDNA deletions, is caused by mutations in *PEO1*, which encodes ‘twinkle’ the putative mitochondrial helicase.^88^ It is thought that twinkle mutations result in an accumulation of replication intermediates, causing replication stalling and eventually depletion. adPEO is also associated with mutations in *ANT1*,^89^ the gene coding adenine nucleotide translocase. Mutations in *ANT1* impair ADP–ATP exchange through the mitochondrial membrane, causing a nucleotide imbalance (affecting replication) and a severe reduction in cellular energy.

In addition to structurally altering mtDNA, several disorders have been identified that are caused by a reduction in mtDNA copy number.^19^ Alpers syndrome, characterized by diffuse and progressive cerebral atrophy,^90^ has been associated with mutations in *POLG*,^91,92^ which cause impairment of the replicative machinery.^93^

Recessive mutations in thymidine phosphorylase cause mitochondrial neurogastrointestinal encephalopathy, characterized by mtDNA depletion, multiple deletions and point mutations. mtDNA depletion has also been identified in early onset hypotonia with myopathy and hepatic involvement, caused by mutations in either thymidine kinase (*TK2*) or deoxyguanosine kinase (*DGUOK*).^94^ Mutations in both of these genes cause a reduction in the mtDNA nucleotide pooling, reducing replication efficiency.

#### Disorders resulting from mutations in nuclear-encoded components or assembly factors of the OXPHOS system

Isolated complex I deficiency is by far the commonest biochemical defect found in mitochondrial disorders; however, it is also the most complex aetiology and clinical spectrum.^95^ Complex I deficiency is associated with a broad range of clinical phenotypes ranging from lethal neonatal disease to adult onset neurodegenerative disorders.^96,97^ A high level of genetic heterogeneity, coupled with weak genotype–phenotype correlations, makes it difficult to predict the genetic basis on pure clinical grounds.^95^ This is important because of the different inheritance patterns and different natural histories of the different genetic causes. However, some patterns are starting to emerge.

There are at least 46 nuclear-encoded subunits of complex I (compared with 7 mtDNA encoded subunits) and so it is unsurprising that nDNA mutations have been identified in 14 of the structural subunits. Pathogenic mutations in *NDUFS1*,^98^ *NDUFS3*,^95,99^ *NDUFS4*,^100^ *NDUFS7*,^101^ *NDUFS8*,^102^ *NDUFV1*,^98,103^ *NDUFA10*,^104^ NDUFB3^95^ and *NDUFA2*^105^ typically manifest as Leigh or Leigh-like syndromes.^60,106^ Conversely, mutations in *NDUFS2*,^107^ *NDUFS6*,^108^ *NDUFV2*,^109^ *NDUFA1*, *NDUFA11*^110^ and *ACAD9*^111^ are typically associated with hypertrophic cardiomyopathy and encephalopathy. In addition, mutations in complex I assembly proteins can manifest as disease: Leigh syndrome (*NDUFAF2* and *NDUFAF5*),^112,113^ encephalopathy (*NDUFAF4*)^114^ and cardioencephalomyopathy (*NDUFAF1*).^115^

Complex II is completely encoded by nDNA and is composed of four polypeptide subunits: *SHD-A*, -*B*, -*C* and -*D*. Mutations in *SHD-A* are rare, but are associated with Leigh's syndrome. Surprisingly, mutations in *SHD-B*, -*C* and -*D* appear to be a common cause of inherited paragagliomas and phaeochromocytomas.^116^

Complex III deficiency typically causes a severe multisystem early onset disorder, which is recessively inherited and rare.^117^^,118^ identified mutations in *BCS1l*, a complex III assembly protein, presenting with neonatal proximal tubulopathy, hepatic involvement and encephalopathy. Subsequently, a deletion in human ubiquinone–cytochrome *c* reductase binding protein of complex III (*UQCRB*) was identified in a consanguineous family presenting with hypoglycaemia and lactic acidosis;^119^ and a missense mutation was identified in *UQCRC*, a ubiquinone-binding protein, in a large consanguineous Israeli-Bedoiun kindred.^120^ More recently, a mutation in *TTC19* (a complex III structural subunit gene) was identified in individuals with a progressive neurodegenerative disorder in late infancy,^121^ expanding the phenotype of complex mutations beyond early infant disorders.

Mutations in complex IV result in severe, typically fatal, infantile disease and to date mutations in four complex IV structural subunits have been identified. A homozygous mutation in *COX6BI*, identified in brothers from a consanguineous Saudi Arabian family, presented with gait instabilities visual disturbances, progressive neurological deterioration and leukodystrophic brain changes.^122^ Mutations in *COX10*, a homologue of yeast haem A:farneslytransferase, are associated with Leigh syndrome^123,124^ and a multisystem disorder.^123^ Atypically, mutations in *COX7B*^125^ are associated with facial dysmorphisms and congenital abnormalities,^126^ and a single mutation in the structural subunit gene, *COX4I2*, was identified in adult Arab Muslim patients with exocrine pancreatic insufficiency, dyserythropoietic anaemia and calvarial hyperostosis.^127^

In contrast, a number of mutations have been identified in complex IV assembly factors. Complex IV assembly gene disorders include *SURF1* (Surfeit locus protein 1), associated with Leigh Syndrome;^128,129^ *C12ORF62* (chromosome 12 open reading frame 62), associated with fatal, neonatal, mitochondrial IV deficiency;^130^ *COA5* (cytochrome *c* oxidase assembly factor 5), associated with neonatal hypertrophic cardiomyopathy^131^ and *FASTKD2*, associated with cytochrome *c* oxidase-defective encephalomyopathy.^132^

Mutations in nDNA-encoded complex V subunit genes also appear very rare. A mutation in *ATP5E* (ATP synthase, H+ transporting, mitochondrial F1 complex, epsilon subunit) was identified in an Austrian woman with complex V deficiency,^133^ and a single gene defect has been identified in the complex V assembly factor gene *ATPAF2*, resulting in impaired complex V activity.^134^

#### Disorders resulting from mutations affecting mitochondrial translation

Several nDNA mutations have been identified which influence the efficiency of mitochondrial translation. Mitochondrial ribosomal protein S16 (*MRPS16*) and mitochondrial ribosomal protein S22 (*MRPS22*) are components of the mitoribosome. Mutations in these genes are known to cause severe, infantile, lactic acidosis, developmental defects in the brain, and facial dysmorphisms (*MRPS16*) and fatal neonatal hypertrophic cardiomyopathy and kidney tubulopathy (*MRPS22*).^135^

Mutations in *PUS1*, peudorine synthase 1, have been shown to cause myopathy, lactic acidosis and sideroblastic anaemia.^136^ The mutation, in the catalytic core of the protein, is thought to disrupt the conversion of uridine to pseudouridine, required for tRNA synthesis.

#### Disorders due to defects in genes controlling mitochondrial network dynamics

Mutations in *OPA1* are primarily a cause of optic atrophy,^66^ but additional phenotypes, such as deafness and neuromuscular disease, have also been seen. Interestingly, mutations in *OPA1* also appear to cause the formation of mtDNA deletions, indicating that Opa1 is also important to mtDNA maintenance.

Much like *OPA1*, defects in *MFN2* cause a disturbance of mtDNA maintenance through impairment of mitochondrial network dynamics.^66^ Mutations in *MFN2* are typically associated with Charcot-Marie-Tooth disease (CMT2A) and hereditary motor and sensory neuropathy (CMT with HMSN type VI).^66^

*DNM1L* (dynamin 1-like), another GTPase, is required for fission of mitochondria.^137^ To date, only a single *DNM1L* has been identified in an infant presenting with both defective mitochondrial and peroxisomal fission.^138^ The patient presented in the first days of life with severe microcephaly, abnormal brain development, optic atrophy with hyperplasia and lactic acidemia.^138^

## Areas of controversy?

### The mitochondrial bottleneck

Mutations in mtDNA are often heteroplasmic, with severity correlating with increasing percentage of mutant. Observations indicate that the amount of a variant inherited from a heteroplasmic mother varies between offspring.^139,140^ This is important when investigating disease aetiology, as an asymptomatic mother, with a sub-clinical heteroplasmy level, can give birth to children with significantly higher levels of an mtDNA mutation.

The ‘mitochondrial bottleneck theory’ attempts to explain this phenomenon.^140^ Briefly, the reduction of mtDNA during early development ‘redistributes’ mtDNA to daughter cells (effectively sharing mtDNA content amongst daughter cells). Oocyte maturation is associated with the rapid replication of mtDNA. This reduction-amplification leads to a purportedly random shift in mtDNA mutational load between cells. Researchers agree that the bottleneck is due to a rapid reduction in mtDNA levels during embryonic development; however, the exact mechanism of segregation is hotly debated. There are currently three leading theories of the mtDNA bottleneck mechanism:^140^ (i) variation in heteroplasmy is due to an unequal segregation of mtDNA during cell division, (ii) variation in heteroplasmy is due to an unequal segregation of mtDNA nucleoids during cell division and (iii) variation in heteroplasmy is due to the selective replication of a specific sub-population of mtDNA.

## Growing points

### Assigning variant causality

Optimal mitochondrial function requires the synergistic cooperation of both mtDNA and nDNA; hence, the investigation of dysfunction requires the interrogation of both genomes. Correctly determining the pathogenicity of potential mutants (in either genome) is critical to understanding mitochondrial disease. This underpins the genetic counselling and subsequent prenatal diagnosis of mitochondrial disorders.

Despite the complexity of both mtDNA point mutations and deletions, as well as the potential for heteroplasmy, assigning pathogenicity to mtDNA variants is analogous to nDNA mutations and is comprehensively described by DiMauro and Schon.^141^ Briefly, the mutation must be present in cases significantly more than asymptomatic controls; if heteroplasmic, the proportion of mutated mtDNA must be higher in patients compared with controls (and subsequently higher in clinically affected tissues compared with unaffected tissues). More importantly, the mutated mtDNA must segregate with defined clinical outcome (described previously). Other criteria, such as evolutionary conservation must be interpreted with care, as very rare neutral variants (so-called ‘private polymorphisms’) or homoplasmic changes (such as in LHON) may be wrongly miss-classified using this approach.^141^ Assigning pathogenicity to tRNA mutations is slightly more challenging; tRNA variants are common; however, a small number of tRNA mutations are responsible for a disproportionate majority of mitochondrial disease.^77^ McFarland *et al*.^77^ provide a comprehensive scoring system which can be used to accurately determine tRNA mutation pathogenicity.

Whole-exome sequencing (WES)^142^ has emerged as the preferred method for identifying Mendelian disease genes, and is proving valuable in the diagnostic evaluation of phenotypically and genetically heterogeneous disorders such as mitochondrial disease.^95,143^ Initially, candidate mutations can be identified by prioritizing known mitochondrial genes, such as the 1500 proposed in ‘MitoCarta’^144^ or Mitop2.^145^ Secondly, WES can drive the discovery of novel mitochondrial disease genes or provide a link to previous disease genes that demonstrate an overlapping clinical phenotype.^146–151^ However, as with all new technologies, care must be taken when interpreting WES data in novel disease genes. Variants identified in poorly characterized genes will require extensive biochemical and functional laboratory analysis to assign causality. Additionally, WES is not wholly comprehensive, not capturing non-coding or regulatory regions and often failing to sequence large portions of the exome.^142,152^ However, as technology improves and bioinformatic analysis becomes streamlined, WES is likely to become a major facet in indentifying nuclear genes that affect mitochondrial function.

### Managing mitochondrial disease

There are limited treatment options for patients with mitochondrial diseases. The main emphasis is on disease prevention and the management of complications. Effective genetic counselling, especially given a family history of mitochondrial disease, is crucial. However, the clinical variability, coupled with the unpredictable inheritance of a heteroplasmic ‘mutant dose’ (through the bottleneck), makes a definite diagnosis difficult.^153,154^

Empiric recurrence risks are available for common homoplasmic mutations (i.e. for LHON), but genetic counselling for heteroplasmic mutations is difficult because of the genetic bottleneck (described earlier). Increased knowledge of the natural history of specific mitochondrial disorders has informed clinical practice. Particular attention to cardiac, ophthalmological and endocrine complications (especially diabetes), can lead to prompt supportive management.^155^ However, there are no specific disease-modifying treatments at present, although some drugs show promise.^156^

An area that has had some *in vitro* and pre-clinical success is the development of ‘gene therapies’.^157^ There are currently three strategies for applying gene therapy to mitochondrial disease: (i) the rescue of an RC defect by expression of a ‘replacement’ gene product from the nucleus (so-called allotopic and xenotpoic expression,^158,159^ (ii) the rescue of a primary mitochondrial defect by importing ‘wild-type’ mtDNA into mitochondria (so-called mtDNA transfection) and (iii) manipulation of the heteroplasmic mtDNA balance (i.e. adjusting the wild-type:mutant type ratio), which can be achieved by improving a patients exercise regime.^160^

More recently, and although in very early stages, allogenic haematopoietic stem cell therapy has been successfully used to treat mitochondrial neurogastrointestinal encephalomyopathy, but associated with high mortality.^161^ Similarly, liver transplants in patients (typically children) suffering from *MPV17*-associated hepatocerebral mitochondrial depletion syndrome have a poor prognosis.^162^

Pre-implantation genetic diagnosis can assist female heteroplasmic mtDNA mutation carriers in determining the risk to their offspring, assisting by preventing transmission of deleterious mtDNA.^163,164^ Briefly, embryos obtained after *in vitro* fertilization are analysed and only those with very low-level mutant levels are transferred to the uterus. However, these techniques are of little help to woman harbouring intermediate-level heteroplasmic mtDNA mutations, where uncertainty regarding the clinical mutation threshold remains.^163^

Advances, harnessing ‘pro-nuclear transfer’, have made significant steps towards treating primary mitochondrial disease at a mtDNA level.^165^ Briefly, the technique involves the transfer of nDNA from a donor zygote (from the mtDNA mutation carrier mother) to an enucleated recipient zygote via fusion. The new ‘reconstructed zygote’ retains the nDNA from the mother, but the mtDNA from a donor. More recently, a competing group has attempted a similar technique, utilizing ‘spindle transfer’ of nDNA to an enucleated donor.^166^ Unlike pro-nuclear transfer, nDNA isolation occurs pre-fertilization, meaning once the technique is approved it can be integrated into established *in vitro* fertilization techniques. However, caution is advised, as both pro-nuclear transfer and spindle transfer would only benefit a minority of female mtDNA mutation carriers, whereas prenatal diagnostic testing can be utilized for both all Mendelian mitochondrial disorders and the majority of mtDNA mutations.^163,167^

## Funding

Funding to pay the Open Access publication charges for this article was provided by The Wellcome Trust.
